# Dynamic Distribution of ASIC1a Channels and Other Proteins within Cells Detected through Fractionation

**DOI:** 10.3390/membranes12040389

**Published:** 2022-03-31

**Authors:** Libia Catalina Salinas Castellanos, Rodolfo Gabriel Gatto, Silvia Adriana Menchón, Matías Blaustein, Osvaldo Daniel Uchitel, Carina Weissmann

**Affiliations:** 1 Instituto de Fisiología Biologia Molecular y Neurociencias (IFIBYNE), Consejo Nacional de Investigaciones Científicas y Técnicas (CONICET), Departamento de Fisiología, Biología Molecular y Celular (DFBMC), University of Buenos Aires (UBA), Buenos Aires 1428, Argentina; licasaca@gmail.com (L.C.S.C.); ouchitel@gmail.com (O.D.U.); 2 Department of Bioengineering, University of Illinois at Chicago, Chicago, IL 60607, USA; rodogatto@gmail.com; 3 IFEG-CONICET and FaMAF-Universidad Nacional de Córdoba, Ciudad Universitaria, Córdoba 5016, Argentina; silmenchon@gmail.com; 4 Departamento de Fisiología, Biología Molecular y Celular (DFBMC), Facultad de Ciencias Exactas y Naturales (FCEyN), Instituto de Biociencias, Biotecnología y Biología Traslacional (iB3), University of Buenos Aires (UBA), Buenos Aires 1428, Argentina; mtsblaustein@gmail.com

**Keywords:** fractionation, plasma-membrane-associated proteins, distribution, translocation, trafficking

## Abstract

Proteins in eukaryotic cells reside in different cell compartments. Many studies require the specific localization of proteins and the detection of any dynamic changes in intracellular protein distribution. There are several methods available for this purpose that rely on the fractionation of the different cell compartments. Fractionation protocols have evolved since the first use of a centrifuge to isolate organelles. In this study, we described a simple method that involves the use of a tabletop centrifuge and different detergents to obtain cell fractions enriched in cytosolic (Cyt), plasma membrane (PM), membranous organelle (MO), and nuclear (Nu) proteins and identify the proteins in each fraction. This method serves to identify transmembrane proteins such as channel subunits as well as PM-embedded or weakly associated proteins. This protocol uses a minute amount of cell material and typical equipment present in laboratories, and it takes approximately 3 h. The process was validated using endogenous and exogenous proteins expressed in the HEK293T cell line that were targeted to each compartment. Using a specific stimulus as a trigger, we showed and quantified the shuttling of a protein channel (ASIC1a, acid sensing ion channel) from the MO fraction to the PM fraction and the shuttling of a kinase from a cytosolic location to a nuclear location.

## 1. Introduction

Tissue fractionation, a process that yields pure samples of the parts of an original whole, has been carried out for a long time. It started with methods that broke up cells and separated, with a centrifuge, some of their parts for the purpose of analysis [1]. The first use of a centrifuge to isolate a cell organelle was by Miescher; it developed further and, in the words of de Duve, turned from a preparative approach to a quantitative approach pioneered by Claude [2]. This approach initially used liver tissue for the quantification of enzymatic activity in the different fractions obtained [1] and constituted a bridge between morphology and biochemistry [3]. The evolution of this methodology, with the use of different equipment, occurred over time and made it possible to gain insight into the different organelles present in cells.

“Classical” fractionation methods based on differential centrifugation require large samples to produce sufficiently concentrated fractions. Instead, methods employing detergents allow working with small sample quantities [4]. Many protocols and commercial kits have been developed for the fractionation of different tissue or cell cultures. The use of digitonin, which solubilizes membranes according to the amount of cholesterol present in the membrane, is seen in some protocols, provided that the amount does not solubilize internal membranous organelles [5,6]. Subsequent steps involve extracting proteins from membranes. Proteins are soluble in micelles formed by amphiphilic detergents. Micelles solubilize membrane proteins by mimicking the natural lipid bilayer environment normally inhabited by proteins [7]. SDS is an ionic traditional surfactant that performs extremely well when solubilizing membrane proteins [8], and its main disadvantage of rendering proteins in a denatured state does not hinder the identification and quantification of proteins when Western blotting is used. Another less common solubilization technique used during the sample preparation process is sonication, and by using this procedure, hydrophobic vesicles are dispersed, releasing membrane protein clusters and allowing them to better interact with detergents in a solution [8].

In this paper, we describe a method based on previous methods that isolates and detects proteins enriched from four different fractions: the cytosol (Cyt), the plasma membrane (PM), membranous organelles (MO), and the nucleus (Nu). Its novelty lies in the isolation of plasma membrane proteins, and not a “membrane fraction” using small amounts of material and common equipment in a lab, without having to use gradients or ultracentrifuges. This can be used for the quantification of a protein of interest in the different fractions or between different conditions. The method takes less than 3 h and was validated with antibodies to endogenous proteins as well as with the expression of exogenous proteins.

We describe how to proceed with the HEK cell line, one of the most common cell lines used for exogenous protein expression. Our method provides a simple and rapid way to start characterizing the localization of exogenous or endogenous proteins within cells and changes in their distribution via stimuli. This method serves to identify the distribution of transmembrane proteins such as channel subunits as well as PM-embedded or weakly associated proteins.

## 2. Materials and Methods

### 2.1. Cellular and Molecular Biology

Human embryonic kidney 293 (HEK) cells (passage 18–26, American Type Culture Collection (ATCC) number CRL-1573) were maintained by serial passages.

For biochemical and molecular analyses, 6- or 12-well plates were coated with 0.1 mg/mL of poly-l-lysine (PLL, Sigma, P2636, St. Louis, MO, USA), and HEK cells were plated at a density of 2.2 × 105 or 1.4 × 105 respectively. HEK cells were grown in Dulbecco’s Modified Eagle’s Medium containing 4 mM L-glutamine, 4.5 g/L glucose, and 110 mL/L sodium pyruvate and supplemented with 10% fetal calf serum (*Natocor*) (or without serum) kept at 37 °C and under 5% CO_2_. Transfection of the cells was performed with the calcium phosphate method as described previously [9] and, specifically for eGFP-ASIC1a, using 1.5 μg DNA of plasmid per 35 mm dish (or 8 cm^2^ growth area). Transfected cells were used 2 days after transfection. For treatments, sorbitol was used at 0.4 M concentration for 20 min at 37 °C.

For whole-cell lysate (WCL) preparation, cells were washed once in PBS and resuspended in a 1% SDS HEPES pH 7.4 lysis buffer containing a protease inhibitor cocktail (Roche, cOmplete) and sonicated (Fisher Scientific Sonic Dismembrator, model 500) (Thermo Fisher, Waltham, MA, USA) at a 20% amplitude for a 15 s pulse.

Protein concentration was estimated with a NanoDrop 2000 (Thermo Fisher).

For microscopy experiments, cells were plated on glass coverslips (12 mm rounded Carolina Assistant-Brand Cover) coated with 1 mg/mL of PLL (Sigma, P2636).

All materials were purchased from Sigma unless stated otherwise.

Plasmids Cells were transfected with the following plasmids: eGFP-C1 plasmid from Clontech; mRFP-PM, a membrane mRFP, a fusion construct made of the PH (pleckstrin homology) domain of phospholipase C δ1 fused to mRFP subcloned from the eGFP version [10] (kind gift from Dr. Scorticati); eGFP-ASIC plasmid (a kind gift from Dr. Gründer); Lck-FRBT2098LKLW-HA-ECFP (CFP-PM) and Tgn38-FRB-HA-ECFP (CFP-TGN-38), which were kind gifts from Vibor Laketa.

### 2.2. Fractionation Assay

The following buffers were used in this procedure in a volume of 125 uL (except for the resuspension of the PM to concentrate in 62.5 μL):

Extraction buffer (EB): NaCl 150 mM, HEPES 50 mM; RIPA buffer: NaCl 150 mM, HEPES 50mM, SDS 1%, sodium deoxycholate 0.5%, pH 7.4.

– To solubilize the cytosolic fraction: digitonin buffer: digitonin 95 μg/mL EB + inhibitor cocktail.
– To solubilize the PM fraction (2% SDS RIPA + inhibitor cocktail).
– To solubilize the MO fraction (1% NP40 EB + inhibitor cocktail).
– To solubilize the Nu fraction (RIPA + inhibitor cocktail).

**Figure 1:**
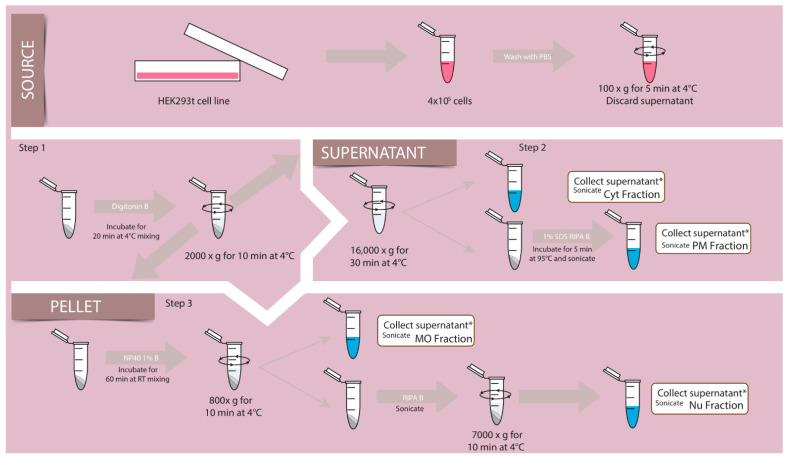
Schematic representation of the fractionation method. HEK293 cells (source) grown on plates were subjected to different steps to obtain the proper cell density to expose them to buffers (B) containing different detergents (arrows) and centrifugation (circle arrow) to obtain different supernatant and pellets enriched in Cyt, PM, MO, Nu fractions. Details in scheme and Material and Methods section. (*) final fractions. Details in scheme and Material and Methods section.

The procedure was as follows (illustrated in Figure 1):

Transfected or untransfected cells were detached from 3.5 cm plates with trypsin to determine cell density. Per assay, 4 × 105 cells were used. To eliminate the incubation medium, cells were washed twice with PBS and centrifuged for 5 min at 100× *g* at 4 °C.

As a first step, the pellet obtained was incubated 20 min with the digitonin buffer, shaking slowly at 4 °C, and then centrifuged at 2000× *g* for 10 min at 4 °C; this yielded a supernatant (I) with the combined Cyt and PM fraction and a pellet (I) which was further processed (see Figure 1, step 1). In a second step, the supernatant was centrifuged at 16,000 *g* for 30 min at 4 °C; the supernatant (I) was separated as Cyt and sonicated, and the pellet resuspended in a 1% SDS RIPA buffer as PM and sonicated (see Figure 1, step 2). In a third step, the pellet (I) was incubated in EB buffer 1% NP40 for 1 h at 4 °C to be centrifuged at 800 *g* for 10 min at 4 °C (see Figure 1, step 3). The supernatant was separated and sonicated and stored as MO fraction. The pellet was incubated with RIPA buffer and sonicated to centrifuge at 7000 *g* for 10 min at 4 °C. The supernatant obtained was sonicated and separated as the Nu fraction. The pellet obtained and resuspended did not show any of the markers of either fraction. All sonication steps were performed on ice with a 20% amplitude pulse for 15 s in the Sonic Dismembrator, model 500 (Fisher Scientific). Laemmli buffer was added to all the fractions for a 1× final dilution and stored at −80 °C until samples were loaded on gels.

### 2.3. Western Blotting (WB)

Proteins were resolved by 4–10% polyacrylamide gels and transferred onto PVDF membranes (Bio-Rad). Nonspecific binding was blocked by 1% nonfat powdered milk in TBS containing 0.2% Tween-20 for 60 min at RT. Membranes were incubated overnight at 4 °C with primary antibodies in 1% BSA TBS, followed by the addition of horseradish-peroxidase-conjugated secondary antibodies in 1% nonfat powdered milk in TBS. Immunoreactive bands were detected by chemiluminescence using horseradish-peroxidase-conjugated secondary antibodies and an ECL detection kit (Bio-Rad, Clarity) following the manufacturer’s instructions. HRP-conjugated secondary antibodies were obtained from Santa Cruz: sc-516102 (anti-mouse), sc-2357 (anti-rabbit). Due to technical problems, some Western blots were detected by the LI-COR Odyssey system, using Immobilon-FL PVDF membranes and secondary antibodies 926-68073 IRDye 680RD Donkey anti-Rabbit IgG or 926-32212 IRDye 800CW Donkey anti-Mouse.

The following primary antibodies were used: rabbit polyclonal anti-ASIC1 (Alomone ASC-014, 1:5000); mouse monoclonal anti-tubulin (DM1a) (Cell Signaling #3873, 1:5000); rabbit polyclonal anti-total ERK (Santa Cruz, C9, 1:500); mouse monoclonal anti-GAPDH (Santa Cruz, sc-365062; 1:1000); GFP (Santa Cruz sc-81045, 1:1000); mouse monoclonal anti-NaKATPase (NaK) alpha (Santa Cruz, sc-58628; 1:1000); mouse monoclonal anti-calnexin (Santa Cruz, sc-23954; 1:1000); mouse monoclonal Anti-H3 (Santa Cruz, sc-56616; 1:1000); mouse monoclonal anti-DsRed (Santa Cruz, sc-390909; 1:1000).

Images were taken using the Fuji AI600 imaging system or LI-COR Odyssey system and quantified with ImageJ software (NIH, New York, NY, USA).

### 2.4. Detection of Proteins by Immunofluorescence (IF)

Cells grown on PLL-coated glass coverslips. They were fixated with 4% p-formaldehyde in PBS, permeabilized with 0.1% Triton x-100 (10 min), and treated with blocking solution (1% BSA, 0.01% Triton x-100 in PBS) for an hour at RT. Coverslips were incubated with the primary antibody for 1 h in blocking buffer, washed in PBS, and incubated with the secondary antibody for 60 min in blocking buffer. After a final wash in PBS, coverslips were placed onto a slide and covered with mounting medium. Propidium iodide (Molecular Probes) was used in a solution at 10 μM in PBS containing Tx-100 (0.01%) and RNAse (20 μM) for 15 min to detect nuclei. DiIC18 (FluoProbes) was used at a 5 μM concentration in PBS solution for 15 min to stain PMs of cells. CNX antibody calnexin (Santa Cruz, sc-23954, Dallas, TX, USA) was used in a 1:100 dilution.

Images were taken using an Olympus FV300/BX61 microscope with 100× and 60× (1.4 NA) oil-immersion objectives. Alexa-647- and Alexa-488-conjugated secondary antibodies, as well as PI and eGFP, were excited using an argon (lambda: 488 nm) laser and helium–neon (lambdas: 543 and 633 nm) lasers and a transmission light detector. Optical sections for stacks were of 2 um/steps. For comparisons, identical laser power and acquisition settings were used. For image processing, images were imported into ImageJ software (NIH).

### 2.5. Data Analysis and Figure Preparation

Data were analyzed by Student’s *t*-test and plotted using GraphPad Prism version 8.01 (GraphPad Software Inc., La Jolla, San Jose, CA, USA). Images were designed using Adobe Illustrator CC version 10 software (Adobe Inc., San Jose, CA, USA).

## 3. Results

We previously described the steps we performed to obtain proteins from four different compartments (Cyt, PM, MO, and Nu) using HEK cells, as schematized in Figure 1.

### 3.1. Solubilization of the PM to Obtain a “Cytosolic Plus” Fraction

**Figure 2:**
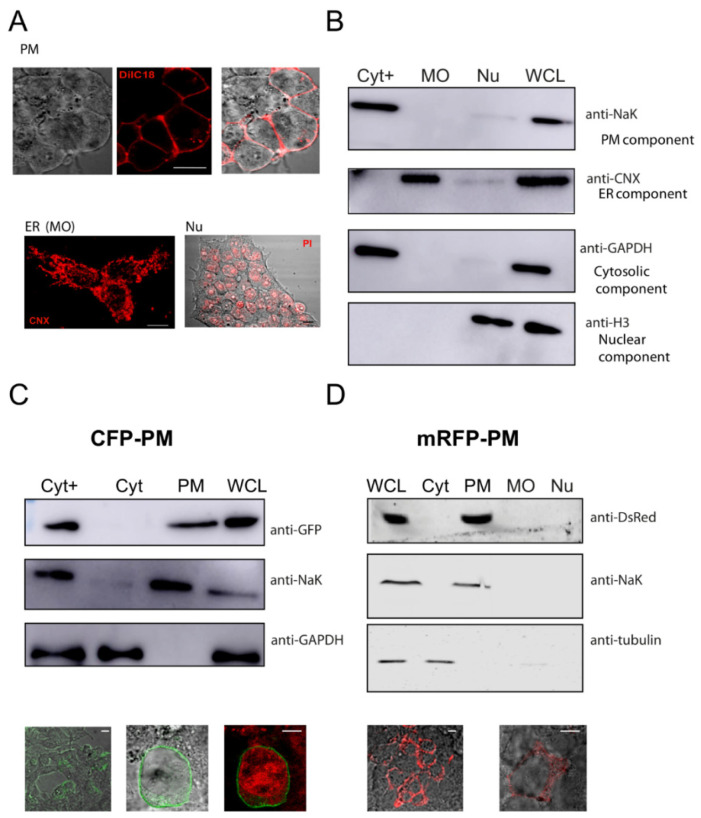
Extraction of proteins from different cell compartments. (**A**) Fluorescent microscopy images of the HEK293 cells used showing the labeling of the plasma membrane (top panel) with DiIC18 dye (red, middle panel), a light microscopy image (left panel), and the merge (right panel). The CNX (calnexin, red) stain (bottom left panel) used as a marker for MO or the PI (Propidium iodide, red) stain (bottom right panel) used as a marker for the nucleus. (**B**) Representative WB membrane showing lysates generated from HEK cells fractionated into a Cyt+ (cytosolic plus fraction), MO (membranous organelle), and nucleus comparing proteins enriched in the fractions to those present in a WCL (whole-cell lysate). (**C**) Membrane showing the extraction of the Cyt and PM fractions from the initial Cyt+ fraction illustrated by the enrichment of the CFP-PM-expressed protein detected by a GFP antibody concomitant with the enrichment of a NaK (sodium–potassium ATPase) antibody, separated from the Cyt (enriched in GAPDH protein with the absence of GFP band); lower panels show CFP-PM- expressing HEK cells images (with transmitted light and PI staining). (**D**) Cells expressing the mRFP-PM protein were extracted to show the protein enrichment in the PM fraction (using an anti-DsRed antibody) separate from all other fractions, with confirmation of the enrichment of NaK in the same fraction; lower panels show images of the cells used detected with filters for mRFP excitation and a light microscopy merged image. Scale bar, 10 µm.

One of our goals was to obtain a fraction enriched in PM proteins. Therefore, the first step of our fractionation protocol was to separate the Nu and MO fractions from a fraction which we called “cytosolic plus” (“Cyt+”) fraction, which comprised Cyt and PM proteins and was from what we aimed at separating PM proteins in HEK cells. The HEK cell line consists of cells with different shapes and a big nuclear compartment (about 2/3 of the cell), as shown in Figure 2A for the different compartments that are stained with markers.

The use of digitonin has been described in previous protocols to permeabilize the PM of cells [5,6]. Digitonin concentration must be properly tuned for the cell type used to solubilize the PM and release cytosolic proteins without the permeabilization of other membranous organelles. Thus, different amounts of digitonin were tested for this purpose. Also, having the proper amount of cells is crucial to obtain initial fractions, especially if cells are transfected and used at different time points after transfections. Therefore, it was important to assess cell density for this initial step. In this work, HEK cells were dislodged in trypsin and counted. A density of 4–5 × 10^5^ cells gave the best results. This amount of cells was washed and then treated with a freshly prepared digitonin-containing extraction buffer (buffer EB) with a concentration of 95 µg/mL, the optimal concentration of the detergent. These initial fractions were used to obtain the solubilized Cyt+ fraction separate from the remaining MO and Nu fractions. The fractions were analyzed by Western blotting to determine the presence of Cyt with cytosolic markers (GAPDH) and MO fractions with ER proteins and an ER marker (calnexin, CNX) (Figure 2B). A marker for the PM (sodium–potassium ATPase, NaK) served to verify that these elements were in the Cyt+ fraction (Figure 2B).

### 3.2. Release of PM Enriched Proteins and Isolation of Cyt and PM Proteins from the Cyt+

We tested for different conditions that would enable the release of PM proteins from the Cyt+ fraction obtained.

To obtain the PM, we tested the combination of a strong surfactant and a sonication step. An SDS buffer and sonication step improved the detection of the integral membrane protein NaK more so than other buffers tested or when sonication was not performed. These solubilized proteins were separatable from the cytosolic proteins.

In our protocol (Figure 1), a simple centrifugation step of the Cyt+ using a tabletop centrifuge was added to separate it into Cyt and PM, relying on the different density of these fractions; then, the step for the release of PM proteins was introduced.

HEK cells transfected with a plasmid encoding for a protein that was myristoylated and palmitoylated, i.e., associated with the PM and containing a CFP tag (“CFP-PM”), were used to test the procedure. Figure 2C shows a comparison of the Cyt+ versus the Cyt and PM enriched fractions. It also shows a whole-cell lysate (WCL) for CFP-PM-transfected cells, i.e., the PM-associated, exogenously expressed proteins that appeared in the Cyt+ fraction and were obtained in the PM fraction after disappearing from the Cyt. Additionally, HEK cells expressing an mRFP-tagged PM protein (mRFP-PM) with a phosphoinositide-binding domain to insert in the plasma membrane were also subjected to the fractionation procedure, which showed the detection of the exogenously expressed protein in the PM fraction and no expression in the other compartments (Figure 2D).

### 3.3. Fractionation of MO and Nu

**Figure 3:**
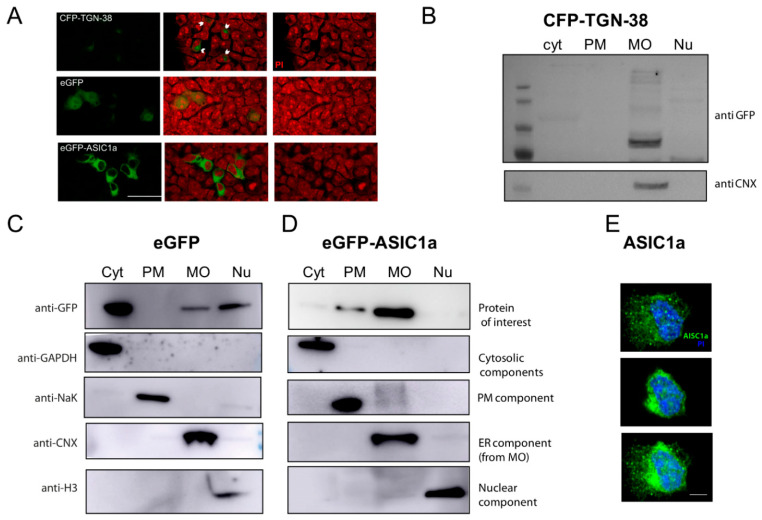
Validation of fractionation method with exogenously expressed proteins. (**A**). Representative immunofluorescence images of HEK cells expressing CFP-TGN-38 (top panels; arrows pointing at areas of cells with eGFP signal), eGFP (middle panels), and eGFP-ASIC1a (lower panels). Signal in green accounted for the fusion protein detected (left panels); PI (Propidium iodide) in red for nuclei (right panels); merge images (middle panel). Scale bar 10 um. (**B**) CFP-TGN-38-expressing cells were subjected to the fractionation protocol, and the lysates for each fraction were analyzed by Western blot experiments. As visualized in a representative membrane, the protein was enriched in the MO fraction (detected with an anti-GFP antibody), as expected, together with a marker for ER (using the anti-calnexin antibody). (**C**) eGFP-expressing cells were subjected to the fractionation protocol, and the lysates for each fraction were analyzed by Western blot experiments. As visualized in a representative membrane, the protein was enriched in the cytosolic, MO, and nuclear fractions, as expected. Each one of the fractions was confirmed to be enriched in proteins known to be abundant (GAPDH for Cyt; NaK for PM; CNX for MO (from the ER); and H3 for Nu). (**D**) eGFP-ASIC1a-expressing cells were subjected to the fractionation protocol, and the lysates for each fraction were analyzed by Western blot experiments. As visualized in a representative membrane, the protein was enriched in the PM and MO fractions. (**E**) Representative confocal microscopy images of HEK293 cells stained with an anti-ASIC1 antibody (and secondary Alexa-488, green), and with PI to visualize nuclei, showing a superficial section (top), a middle section (middle), and a merge of layers (bottom). Notice a predominant perinuclear staining. Scale bar 5 µm.

The cell material obtained after the separation of the Cyt+ fraction was further processed via solubilization with an NP-40 treatment, a nonionic detergent that solubilizes the nuclear membrane, and centrifugation to obtain a supernatant (MO fraction) that was separated from the Nu as described in our scheme (Figure 1). In addition, a plasmid encoding for CFP-tagged-TG-38 (Trans-Golgi network integral membrane protein 38, Figure 3A) was introduced in HEK cells, and the fractionation protocol showed the enrichment of this protein in the MO fraction together with calnexin (Figure 3B–D).

**Figure 4:**
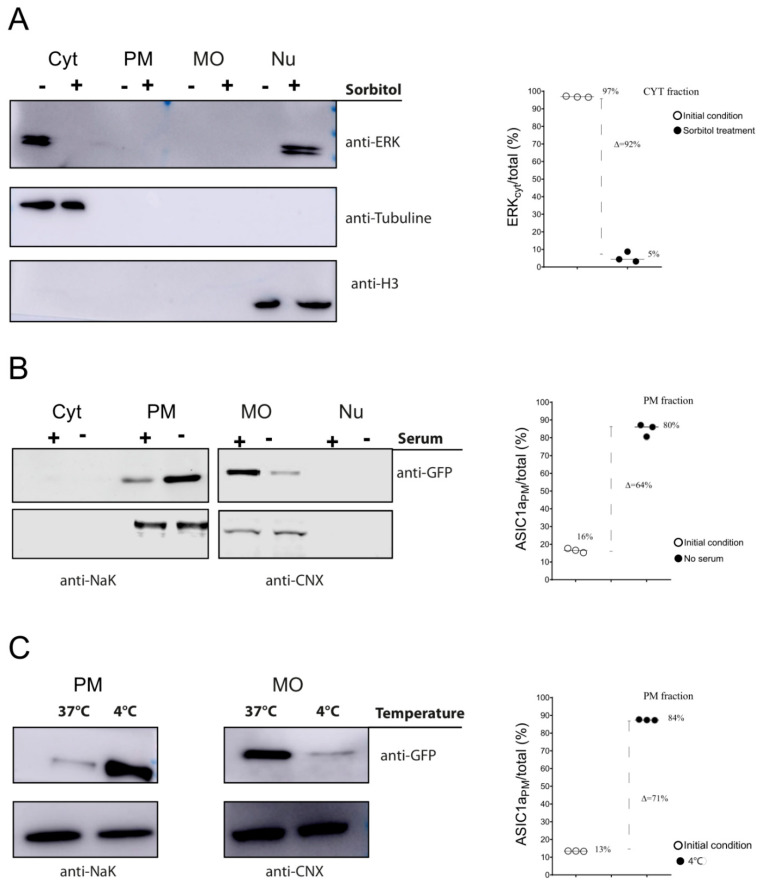
Fractionation to detect changes in protein distribution. (**A**) Representative WB membranes of HEK293 cell lysates obtained by the fractionation method to detect total ERK protein in the different fractions (Cyt, PM, MO, Nu) in control (−) or sorbitol-treated (+) cells using an anti-total ERK antibody with tubulin as a cytosolic marker or histone as a nuclear marker. Plot showing the percent changes of fractions for both conditions. (**B**,**C**) Representative Western blots of eGFP-ASIC1a-HEK293-expressing cell lysates obtained by our fractionation method to detect eGFP-ASIC1a protein in MO and Nu fractions exposed to either a 120 min medium without serum incubation, as opposed to the normal (+ serum) incubation, (**B**) or a 20 min 4 °C incubation, as opposed to the normal 37 °C incubation (**C**); to the right, plots showing changes in the percent fractions for either condition.

The presence of nuclear proteins within the pellet was obtained as Nu was ascertained via the use of a nuclear marker, histone (Figure 2, Figure 3 and Figure 4). The whole procedure was performed on eGFP-transfected cells to visualize the GFP in the cytosolic, MO, and nuclear compartments (Figure 3A,C).

With this method, we obtained an extraction of proteins of approximately 80%, calculated for the protein present in a WCL compared to the sum of proteins in the obtained fractions (based on the concentration of proteins and volumes in the fractions using a nanodrop).

Finally, another exogenously expressed protein was tested with this protocol: the ASIC1a channel. There are several isoforms of ASIC channels [11]. Overexpressed ASIC1 and ASIC2 in heterologous systems predominantly localize to the ER. GFP-ASIC1 and GFP-ASIC2a in cells show a reticular distribution pattern with slight membrane staining, suggesting a predominant localization to the ER [12]. Thus, as documented, the channel should be enriched in the MO and PM fractions [13]. The eGFP-ASIC1a fusion protein expressed in HEK cells [14], Figure 3A, was fractionated and detected as enriched in the fractions expected (Figure 3D). The level at which the protein is expressed is key for its proper distribution (Supplementary Figure S1A) and should be considered, in addition to checking that exogenously expressed proteins are functional. As evident from Figure 3D, the amount of protein present at the PM was much lower than that at the MO. This also became apparent for the endogenously expressed channel, as shown in Figure 3E, by confocal fluorescent images of HEK cells, consistent with the literature.

### 3.4. Dynamic Changes between Compartments

#### 3.4.1. Translocation of a Kinase from the Cytosol to the Nucleus

We used our method to analyze the change in the location of the extracellular signal-regulated kinases 1/2 (ERK) that operate within signaling cascades that transmit extracellular signals to their intracellular targets [15]. In response to different stimuli, this kinase is activated via phosphorylation and translocates to the nucleus [16]. Other methods have been used to check and quantify the translocation of molecules to a Cyt or Nu fraction only [17]. We tested our fractionation method by using sorbitol to induce hyperosmolar stress [18], ERK activation, and translocation to the nucleus. Other methods have been used to check and quantify the translocation of molecules to a Cyt or Nu fraction only [19]. We tested our fractionation method by using sorbitol to induce hyperosmolar stress [20], ERK activation, and translocation to the nucleus.

HEK cells were exposed to sorbitol (0.4 M) for 20 min and fractionated according to our protocol. The different fractions obtained for each condition (control or sorbitol-treated cells) were subjected to Western blot assays and total ERK was detected. As demonstrated in Figure 4A, in normal conditions, most of the total ERK was present in the cytosol fraction, but in response to sorbitol, the amount in the Cyt decreased and that in the Nu increased. The quantification of these bands (see Supplementary information for more details), detected on membranes, showed the almost total translocation of the Cyt ERK to the nuclear fraction.

#### 3.4.2. Channel Trafficking

We used our method to visualize the compartmentalization of ASIC1a channels (Figure 4B,C), and their dynamic changes. The trafficking of this channel has been extensively reviewed [21,22]. In addition, ASIC1a expressed in cells shuttled from a predominant ER location and outer layer nuclear membrane to a PM location after incubation with cell media lacking serum [13]. We tested if this redistribution of the channel, by the incubation of cells in media lacking serum, as used by Chai et al., could be properly detected via our fractionation protocol and if the increase in channels at the PM corresponded to a decrease in channels at the MO.

Figure 4B shows that the initial 16% of PM channel, in the presence of serum, changed to an 80% of the channel at the PM.

Exposure of cells to ice-cold medium (0–4 °C) is a commonly used method for the nonspecific inhibition of endocytosis [20]. We tested how the inhibition of endocytosis via a decrease in temperature affected the channel distribution, changing the predominant channel fraction present in the MO. Fractionation showed how the distribution of the channel changed from the predominant MO to the PM fraction via incubation of the cells at 4 °C (Figure 4C).

This demonstrated the finely tuned regulation of ASIC1a, which, at normal conditions, is present at very low levels at the plasma membrane but can quickly change its location in response to a trigger.

## 4. Discussion

The simple procedure described allowed us to isolate and detect proteins enriched in different fractions pertaining to different cell compartments. Additionally, we noted the changes detected in proteins’ distributions after different stimuli were quantified to compare different conditions. Thus, the translocation of a kinase and the trafficking of a channel were demonstrated and quantified.

Many methods have been described in the literature to fractionate tissue or cells and isolate subcellular components. Among those using minute amounts of starting material and a quick methodology, Baghirova et al., based on previous methodology applied by Holden et al. [5], used gentle buffers with increasing detergent strength that sequentially lysed the cell membrane, organelle membranes, and the nuclear membrane [21]. We extended this method by using a defined low amount of starting cell density (especially relevant when starting from transfected cells); we used a sonication step to release membrane-bound proteins, obtain a PM fraction, and shear DNA strands in the nuclear fraction. We described a methodology to quantify and account for the presence of proteins in their different locations and their redistribution by given cues.

Different examples in the literature showed the redistribution of proteins between compartments, as detected via microscopy in a qualitative manner. To compare how different effects alter the distribution, a quantitative parameter of this fraction is required. Even though biotinylation assays quantitate the amount of protein at the surface, they do not reveal the corresponding direct increases or decreases or which starting compartment they originated from.

In the history of tissue fractionation, what turned the method from a preparative to analytical one was the possibility of accounting for the levels of proteins, i.e., enzymatic activity, in each fraction to obtain a “balance sheet” [3]. We attempted to account for the levels of proteins in each fraction. Accordingly, we compared between the same fractions in different conditions and between the fractions themselves.

Previous work using a fractionation method to separate cytosolic and nuclear proteins in COS-7 cells after serum stimulation caused a substantial shift in ERK to the nucleus (80%) [22]. Plotnikov et al. analyzed ERK nuclear translocation and documented that the majority of resting HeLa cells showed that ERK molecules were localized to the cytosol; however, upon stimulation, immunofluorescent assays showed that most ERK staining shifted to the nucleus (with an approximate sevenfold nuclear increase). A fractionation assay to obtain cytosolic and nuclear fractions confirmed their results [23]. Liu et al. [24] compared several kits and techniques used in fractionation experiments and proposed a methodology to obtain different fractions with different detergents and a density gradient ultracentrifugation step, emphasizing the use of markers for each fraction to validate each experiment. They also analyzed ERK activation and redistribution in L cells treated with an activator of PKC. However, discrepant results were shown between their immunofluorescence and fractionation assays, and no changes in total ERK redistribution were visualized; only phosphorylated ERK was detected as increasing in most fractions. In our case, by using a drastic treatment of sorbitol to induce hyperosmolar stress, we detected an increase in nuclear ERK and its redistribution from the Cyt to the Nu enriched fraction. This Nu fraction was heavily phosphorylated, as has been documented before and shown in Supplementary Figure S1C. The importance of ERK in signaling cascades has been widely studied as a master switch [25] and player involved in the downstream signaling of ASIC1a activation [26]. This work provides additional tools to further characterize these mechanisms.

Apart from obtaining fractions enriched in Cyt and Nu proteins, the methodology described in this work also allowed us to detect the redistribution of a channel in MO and PM enriched compartments. Our methodology showed similar values compared to the biotinylation levels previously reported of a 2.5-fold increase in the amounts of channels at the surface from their initial levels by Chai et al. [13]. Biotinylation encounters some difficulties as not every protein can be labeled on the cell surface due to the lack or inaccessibility of carboxyl and primary amino groups in the extracellular domains [27]; also, this methodology does not allow visualization of the fraction that accounts for the change in the surface levels (as shown in this work, the protein levels decreased in the MO fraction and increased at the PM, Figure 4B).

Assays detecting the increase in channels at the PM via the inhibition of endocytosis showed drastic changes. These changes were amenable to kinetic studies, as the redistribution could be determined at different time points, as shown in Supplementary Figure S1B.

Incidentally, we also determined the importance of the amount of plasmids expressed through transfection methods because too high of an amount, as normally used in many protocols for transient transfections, leads to a high amount of protein detected in the PM, which might also be functional. This is detrimental because it is also recovered in the nuclear fraction (Supplementary Figure S1A), which might be the result of folding disturbances (overexpressed proteins may come down in the nuclear fraction as misfolded proteins that reside in the ER and tend to stick).

A limitation associated with the methodology described is that proteins cannot be further used if analysis of their function is required, i.e., the aim is not to obtain proteins in a native state. Also, it is important to verify detergent concentrations initially to set up the procedure for using markers for each compartment. The steps described are the results of multiple trials in which different cell densities, volumes, times and concentrations for incubation with detergents, and centrifugation parameters were tested. Additionally, the methodology is intrinsically limited by the sensibility of the tools used for the detection of the proteins under analysis: the equipment and the antibodies.

All in all, we described a quick and robust method to detect and quantify proteins enriched in different cell fractions, which is particularly useful for transmembrane proteins, such as channels; we also showed different examples in which the methodology is of great use.

## Data Availability

Not applicable.

## Supplementary Materials

Supplementary Materials are available online at https://www.mdpi.com/article/10.3390/membranes12040389/s1. Figure S1: These experiments were performed twice, and a representative membrane is shown for each, Table S1: Reagents and suppliers used.
