# Supplementary material for: Dynamic Distribution of ASIC1a Channels and Other Proteins within Cells Detected through Fractionation
Source: Membranes (Basel). 2022 Mar 31;12(4):389. doi: 10.3390/membranes12040389 (PMC9027401; doi:10.3390/membranes12040389)
Supplement: Supplementary file 1 [file membranes-12-00389-s001.zip › membranes-1621317-supplementary.pdf]

*membranes*

*Supplemetary Materials*

# Dynamic Distribution of ASIC1a Channels and Other Proteins within Cells Detected through Fractionation

**Libia Catalina Salinas Castellanos [1], Rodolfo Gabriel Gatto [2], Silvia Adriana Menchón [3], Matías Blaustein [4], Osvaldo Daniel Uchitel [1] and Carina Weissmann [1,*]**

[1] Instituto de Fisiología Biologia Molecular y Neurociencias (IFIBYNE), Consejo Nacional de Investigaciones Científicas y Técnicas (CONICET), Departamento de Fisiología, Biología Molecular y Celular (DFBMC), University of Buenos Aires (UBA), Buenos Aires 1428, Argentina; licasaca@gmail.com (L.C.S.C.); ouchitel@gmail.com (O.D.U.)
[2] Department of Bioengineering, University of Illinois at Chicago, Chicago, IL 60607, USA; rodogatto@gmail.com
[3] IFEG-CONICET and FaMAF-Universidad Nacional de Córdoba, Ciudad Universitaria, Córdoba 5016, Argentina; silmenchon@gmail.com
[4] Departamento de Fisiología, Biología Molecular y Celular (DFBMC), Facultad de Ciencias Exactas y Naturales (FCEyN), Instituto de Biociencias, Biotecnología y Biología Traslacional (iB3), University of Buenos Aires (UBA), Buenos Aires 1428, Argentina; mtsblaustein@gmail.com
* Correspondence: carina.weissmann@gmail.com

**Citation:** Salinas Castellanos, L.C.; Gatto, R.G.; Menchón, S.A.; Blaustein, M.; Uchitel, O.D.; Weissmann, C. Dynamic Distribution of ASIC1a Channels and Other Proteins within Cells Detected through Fractionation. *Membranes* **2022**, *12*, 389. https://doi.org/10.3390/membranes12040389

Academic Editor: Arwyn Tomos Jones

Received: 16 February 2022
Accepted: 25 March 2022
Published: 31 March 2022

**Publisher's Note:** MDPI stays neutral with regard to jurisdictional claims in published maps and institutional affiliations.

**Copyright:** © 2022 by the authors. Licensee MDPI, Basel, Switzerland. This article is an open access article distributed under the terms and conditions of the Creative Commons Attribution (CC BY) license (https://creativecommons.org/licenses/by/4.0/).

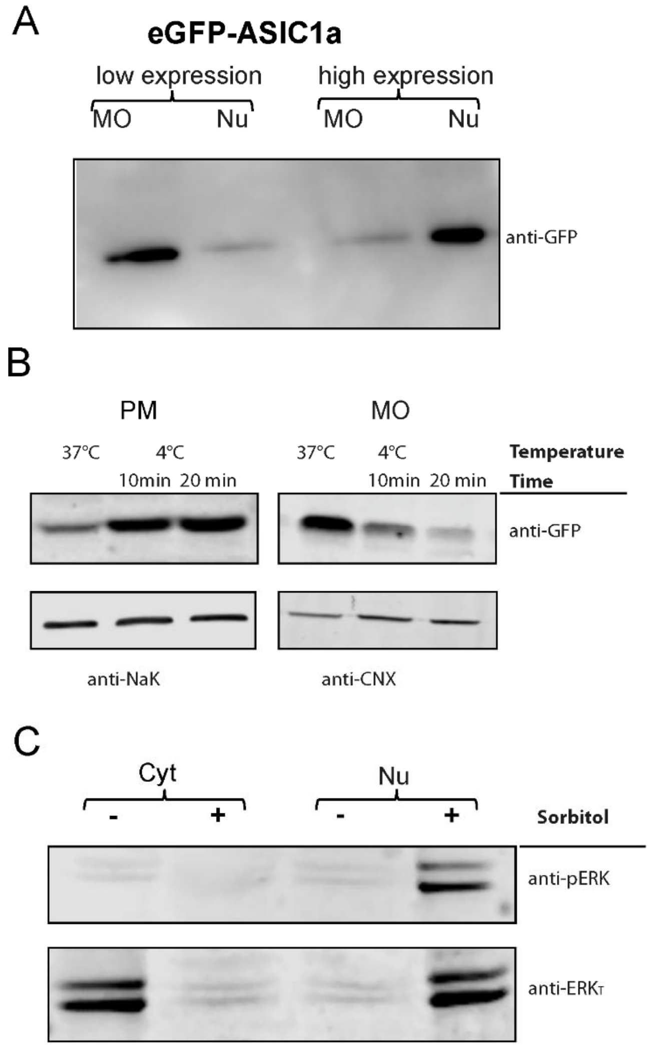

**Figure S1.** These experiments were performed twice, and a representative membrane is shown for each. (**A**) Representative western blot membrane of lysates from eGFP-ASIC1a expressing HEK293 cells subjected to the fractionation protocol to show amounts of eGFP-ASIC1a protein (detected with an anti-GFP antibody) in the MO and Nu compartment for transfections with a low or high amount of plasmid. Notice the abnormal distribution of the protein when high levels of proteins are obtained possibly pointing to abnormal folding of the protein and retention in a nuclear enriched compartment. (**B**) Representative western blot of eGFP-ASIC1a expressing HEK293 cells exposed to a 4 °C incubation for 10 or 20 min and subjected to the fractionation protocol to assess the amount of eGFP-ASIC1a protein in the different fractions shown (PM, OM). (**C**) Representative western blot membrane of lysates from HEK293 cells subjected to the fractionation protocol to show amounts of phosphorylated ERK after treatment with sorbitol in the Nu fraction (top, detected with an antibody against phosphorylated ERK), the fraction in which total ERK increases (lower panel) (detected with an anti-total ERK antibody) as shown in the main text.

**Quantification of Proteins in the Different Fractions**

To be able to compare the different fractions obtained within experiments and between different conditions, some normalization is needed. The turning point of fractionation methods came when, as Duve put into words, the method was transformed from a preparative to a quantitative analytical approach. This was a great contribution of Albert in the field of enzymes: "to express the enzymatic activity exhibited by each fraction in terms of the total activity possessed by the unfractionated liver extract". Thereafter, researchers focused on the importance and "need of establishing balance sheets in which the summation of the activities of the tissue fractions is compared with that of the whole tissue" (C. De Duve & Beaufay, 1981). In the same, way, through this approach, we attempted to quantify the expression of the protein of interest in each fraction as in relation to the sum of all fractions (total). For this reason, the fractions are resuspended in equal volumes. However, in the case of the PM fraction, in order to detect via WB detection methods, the volume had to be concentrated. This is explained as compartments differ greatly in volume (illustrated in Figure 2, with a PM staining by DiIC18 dye, and compared to the ER, exemplified by the staining of CNX marker for ER in HEK cells). In addition, membrane proteins are usually present at low levels in biological membranes (Seddon et al., 2004) (as exemplified with the detection of a channel at the PM, Section 3.4.2).

Comparisons of the same fractions between different conditions are simple, due to the fact that ~~as~~ the markers in each fraction can be used to obtain a normalized quantity (Hay et al., 2019), e.g., the normalized amount of the protein expression in the cytosol is given by:

$$r_1 = \frac{Cytx}{CytCtr}, \quad \text{(S1)}$$

Where *Cytx* represents the amount of protein expression in the cytosol and *CytCtr* denotes the amount of control protein expression in the cytosol. Similarly, $r_2$ can be defined for the nuclear fraction, i.e,

$$r_2 = \frac{Nux}{NuCtr}.$$

Since there is no marker present in all fractions, direct comparisons between $r_1$ and $r_2$ are not accurate. However, we are interested in the portion of protein that is present in different fractions. Thus, in order to compare different fractions obtained under the same conditions (eg, treated cells), the following normalized quantities are used:

$$\frac{Cytx}{Cytx+Nux}, \text{ and } \frac{Nux}{Cytx+Nux}.$$

These quotients yield numbers between zero and one. It is worth noticing that it is possible to define other dimensionless quantities, for instance

$$\frac{Cytx/CytCtr}{(Cytx+Nux)/(CytCtr+NuCtr)}, \text{ and } \frac{Nux/NuCtr}{(Cytx+Nux)/(CytCtr+NuCtr)},$$

and compute their quotients. However, this is not convenient because these numbers are not necessarily in the interval zero and one, and they do not give much more detail, since control protein ratios may be considered invariant between experiments, i.e.,

$$\frac{CytCtr}{CytCtr+NuCtr}, \text{ and } \frac{NuCtr}{CytCtr+NuCtr}$$

can be considered constants.

**Table S1.** Reagents and suppliers used:.

| Reagent | Supplier |
|---|---|
| Digitonin (D141) | Sigma |
| NP-40 (85125) | Thermo Scientific |
| SDS (151-21-3) | Sigma |
| Tween-20 (P7949) | Sigma |
| BSA (I100-10) | Innovative Research |
| HEPES (RU 1907) | Genbiotech |
| PVDF (1620177)& (Immobilon®-FL) | Biorad |
| DiIC18 | FluoProbes |
| ASIC1 antibody (ASC-014) | Alomone |
| DM1a antibody #3873 | Cell signaling |
| total ERK (C9) | Santa Cruz |
| GAPDH sc-365062 | Santa Cruz, |
| GFP sc-81045 | Santa Cruz |
| NaKATPase (NaK) alpha sc-58628 | Santa Cruz |
| calnexin sc-23954 | Santa Cruz |
| H3 sc-56616 | Santa Cruz |
| DsRed sc-390909 | Santa Cruz |
| HRP-conjugated secondary sc-516102 (anti-mouse); sc-2357 (anti-rabbit). | Santa Cruz |
| secondary antibodies: 926-68073 IRDye 680RD Donkey anti-Rabbit IgG or 926-32212 IRDye 800CW Donkey anti-Mouse | Licor |
| protease inhibitor cocktail (cOmplete™) | Roche |
